# Immune profiling of canine B cell lymphoma reveals cross-species conservation of prognostic markers

**DOI:** 10.1038/s41598-025-13389-2

**Published:** 2025-08-04

**Authors:** Dillon Didehvar, Jennifer A. Lenz, Brandon Peng, Audrey Ghanian, Lang Jiang, Matthew J. Atherton

**Affiliations:** 1 Department of Clinical Sciences and Advanced Medicine, School of Veterinary Medicine, University of Pennsylvania, Philadelphia, PA USA; 2 Department of Biomedical Sciences, School of Veterinary Medicine, University of Pennsylvania, Philadelphia, PA USA

**Keywords:** Comparative oncology, B cell lymphoma, Immune contexture, Angiogenesis, Cancer, Non-hodgkin lymphoma

## Abstract

Only 60% of patients with diffuse large B cell lymphoma are cured following standard of care therapies. While immune contexture is associated with outcomes in patients treated with chemotherapy, immune mechanisms driving differential therapeutic responses remain unclear. Here, we undertook a comparative analysis of dogs with spontaneous B cell lymphoma (BCL), which exhibit similar dichotomies in therapeutic outcome, to identify conserved and species-specific transcriptional and circulating biomarkers associated with remission duration. In addition, we compared treatment naive and relapsed samples to determine how treatment impacts immune contexture at the time of treatment failure. Among eighteen client-owned dogs with aggressive BCL undergoing multi-agent chemotherapy, comparative immune profiling revealed increased T cell transcripts associated with prolonged remissions and, as in humans, *IL2RB* expression was associated with favorable outcomes. Increased angiogenic markers were associated with shorter remissions. In treatment naive samples, macrophage associated cytokines were increased, whereas multiple T cell-associated transcripts were enriched in relapsed nodes. Collectively, our findings reveal that changes in immune composition are associated with varying chemotherapeutic outcomes in canine BCL and highlight the potential for comparative oncology approaches to identify factors associated with disease progression, providing insight for development and testing of novel therapeutic approaches.

**Supplementary Information:**

The online version contains supplementary material available at 10.1038/s41598-025-13389-2.

## Introduction

Non-Hodgkin lymphomas (NHL) represent a significant cause of morbidity and mortality, and it is anticipated that 80,000 new cases and 19,000 deaths from NHL will occur in the United States in 2025^1^. Diffuse large B cell lymphoma (DLBCL) represents the most common subtype of NHL in people^2–4^. The current standard of care for DLBCL consists of the anti-CD20 antibody rituximab combined with cyclophosphamide, doxorubicin, vincristine, and prednisone (R-CHOP)^3–5^. Although approximately 60% of patients will be cured by R-CHOP, the significant number of treatment failures highlights the critical need to further understand the factors associated with outcomes to ultimately improve therapy^4^.

Cytotoxic chemotherapeutic regimens can be profoundly immunosuppressive, however, their somewhat counterintuitive ability to differentially engage antitumor immune responses in patients influences clinical outcome^6,7^. For example, in DLBCL patients who were cured following CHOP regimens, there was an enrichment of T cell signatures compared to patients with progressive disease^8^. A recent study similarly revealed that immunologically depleted tumor microenvironments (TMEs) were associated with poor outcomes in DLBCL^9^. Collectively, these studies highlight the importance of immune status in shaping therapeutic outcomes in DLBCL and suggests that at-risk patients exhibiting less favorable immune signatures might benefit from additional treatments to favorably shift their immune response, thereby improving survival.

The field of comparative oncology offers a unique opportunity to further study the biology of malignancies^10^. Notably, this approach has identified canine cancer patients as a particularly valuable model, as dogs are genetically outbred, immunologically intact, and develop spontaneous tumors that can closely emulate those of their human counterparts^11–13^. Lymphoma is the most frequently encountered hematologic malignancy in dogs and DLBCL is the most common subtype, accounting for around 40% of all canine lymphoma^14–17^. At the genetic level there is significant overlap between human and canine DLBCL, and multi-agent CHOP chemotherapy is considered the mainstay of first line care for aggressive canine B cell lymphoma (BCL)^15,16,18–21^. Despite favorable initial responses to chemotherapy, remission is often short lived for aggressive BCL with most dogs succumbing to disease within 10–14 months^22,23^. Interestingly, whilst a subset of dogs fails to respond to the CHOP regimen, other dogs exhibit prolonged survivals of greater than two years^22,23^. Although transcriptional changes in the TME have been found to be associated with outcome in canine BCL patients undergoing experimental combination immunotherapy approaches, there is a paucity of prospective literature evaluating potential immune biomarkers for dogs undergoing standard cytotoxic CHOP treatment^24,25^. As dogs with spontaneous BCL share multiple genetic and biological similarities with human BCL patients, we posited that a comparative approach would reveal conserved markers associated with outcome across the species barrier and could potentially reveal differences that might be exploited to improve outcomes in both species^15,18–21^.

In this study, we established a minimally invasive biobank to preserve samples from prospectively enrolled canine patients diagnosed with aggressive BCL undergoing CHOP treatment. Our primary objective was to screen for transcriptional and circulatory markers associated with outcome following CHOP chemotherapy. A secondary objective aimed to document changes in immunity in dogs with BCL between time of diagnosis and first relapse. We found that markers associated with angiogenesis were enriched in dogs with short remissions whereas multiple T cell transcripts, including *IL2RB*, were enriched in dogs with exceptional responses to CHOP, the latter of which is also associated with prolonged survival in human DLBCL patients. These findings provide rationale to further study how angiogenesis and T cell mediated immunity shape outcomes in canine BCL with a view toward trialing novel approaches that target these features in spontaneous canine lymphoma for the benefit of both species.

## Materials and methods

### Trial design and inclusion criteria

**Fig. 1:**
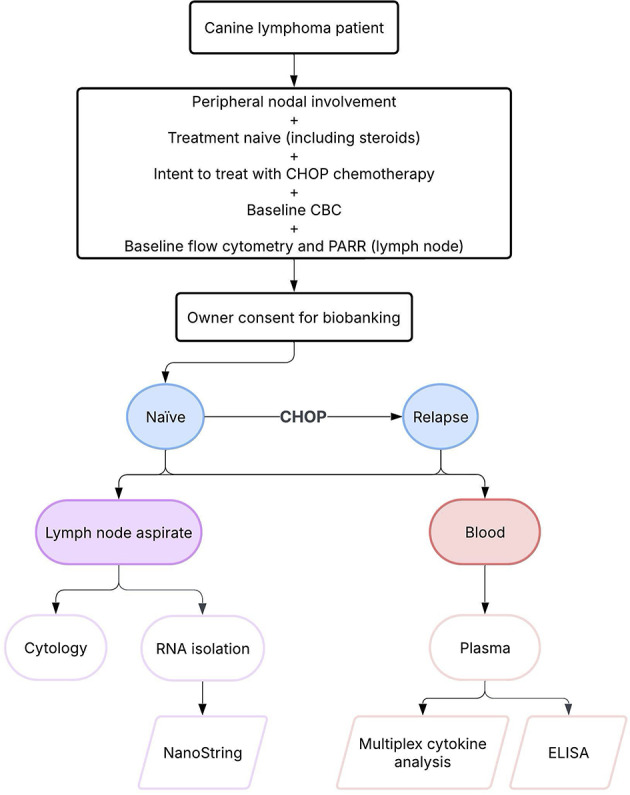
Schema for prospective enrollment of dogs undergoing CHOP treatment for minimally invasive immune profiling of aggressive BCL.

Dogs were recruited prospectively from November 2021 to March 2023 at the University of Pennsylvania School of Veterinary Medicine. Dogs diagnosed by cytology with intermediate-to-large cell lymphoma that had not received prior treatment, including steroids, were eligible for enrollment. BCL was confirmed by flow cytometry and PCR for antigen receptor rearrangement (PARR). Multicentric presentation (enlargement of at least 2 peripheral lymph nodes) was required. Dogs with concurrent or prior neoplasia, and/or life-threatening comorbid disease were excluded. All dogs were enrolled with the intention to complete a 19-week CHOP protocol (Table S1). Initiation with L-asparaginase at the beginning of CHOP was allowed. All dogs had a baseline complete blood count (CBC), with further staging performed at the discretion of the attending veterinarian. Lymph node fine needle aspirates (FNAs) and blood samples were collected at baseline (before treatment initiation) and at relapse (Fig. 1). Response to therapy was assessed using caliper measurements of peripheral nodes to determine remission status in line with the Veterinary Cooperative Oncology Group (VCOG) response evaluation criteria for peripheral nodal lymphoma in dogs^26^. Repeat CBCs were routinely performed prior to the administration of each dose of cytotoxic agent to ensure white cell counts were adequate. CBCs were also routinely performed at the one-week nadir following doxorubicin in the first two cycles of CHOP, and nadir samples were subsequently performed at the discretion of the attending veterinarian in the last two cycles of CHOP. Toxicities were scored according to published VCOG guidelines and doses were modified at the attending veterinarian’s discretion if required^27^. The minimum database collected from each dog included signalment, weight, immunophenotype, baseline blood counts, minimum stage, substage, drug dosage and administration dates, treatment response status at each visit, date of progression and date of death. When necessary, further information was obtained by contacting referring veterinarians and owners. This study was approved by and performed in accordance with the University of Pennsylvania’s Institutional Animal Care and Use Committee (IACUC, #806,853), and the Privately Owned Animal Protocol committee (POAP, #589). Signed consent to enroll in this study was obtained from the dog owners.

### Diagnostic CBCs, cytology, flow cytometry, and PARR

Cytology slides were stained in Wright-Giemsa and prospectively evaluated by a board-certified clinical pathologist to confirm diagnosis. B cell lineage was confirmed via flow cytometry and PARR testing at the Colorado State Clinical Hematopathology Laboratory, as described^28,29^. Baseline CBCs were run on a Siemens Advia 2120 Hematology Analyzer (Siemens Medical Solutions, Malvern, PA, USA).

### Sample processing and storage

Samples were acquired from canine lymphoma patients within one day prior to starting CHOP for naive samples and at time of relapse. Cells for subsequent RNA extraction were acquired using FNAs of neoplastic nodes and collected into citrate tubes (Sarstedt Inc, Newton, NC, USA). Cells were washed once with PBS, pelleted, lysed in 1mL TRIzol Reagent (Thermo Fisher Scientific, Waltham, MA, USA), and these solutions were cryopreserved at -80 °C until RNA isolation. Whole blood was collected by venipuncture into potassium EDTA tubes (Cardinal Health Inc, Dublin, OH, USA). Anticoagulated blood was centrifuged at 2000 g for 15 min at room temperature prior to collection of plasma samples that were stored at -80 °C prior to use.

### RNA isolation

Cryopreserved lysates were incubated at room temperature for 5 min before addition of 200µL of chloroform (Sigma-Aldrich, St Louis, MO, USA) and vortexed. Solutions were then centrifuged immediately at 12,000 g for 5 min at 4 °C. The aqueous RNA containing phase was transferred to a fresh microcentrifuge tube prior to the addition of 1 volume of 70% ethanol. Samples were loaded onto RNeasy mini columns and RNA was isolated following the manufacturers protocol (RNeasy Mini Kit, Qiagen Sciences Inc, Germantown, MD, USA). RNA concentrations were determined using a Qubit 4 Fluorometer (Thermo Fisher Scientific, Waltham, MA, USA) and DV200 values were calculated using Tapestation (Agilent Technologies, Santa Clara, CA, USA). Purified RNA was stored at -80 °C until NanoString profiling.

### NanoString transcriptional profiling

A 5µL solution of 100ngs of RNA was hybridized with gene specific reporter and capture probes (nCounter Canine IO panel, NanoString, Seattle, WA, USA) at 65 °C for 18 h and processed on the nCounter Prep station. Data was acquired using the nCounter scanner, both systems are part of the NanoString nCounter Flex system. NanoString data were analyzed by ROSALIND (https://rosalind.bio/*)*, with a HyperScale architecture developed by ROSALIND, Inc. Normalization, fold changes, and p values were calculated using criteria provided by NanoString. ROSALIND follows the nCounter Advanced Analysis protocol of dividing counts within a lane by the geometric mean of the normalizer probes from the same lane. Housekeeping probes to be used for normalization were selected based on the geNorm algorithm as implemented in the NormqPCR R library^30^. Clustering of genes for the final heatmap of differentially expressed genes was done using the PAM (Partitioning Around Medoids) method using the fpc R library (https://cran.r-project.org/web/packages/fpc/index.html) that takes into consideration the direction and type of all signals on a pathway, the position, role, and type of every gene. When comparing naive and relapsed samples a unique patient identifier was included as a covariate to account for sample pairing. Differentially expressed genes were reported when fold change was ≥ 1.5 or ≤-1.5 and were considered statistically significant when p < 0.05.

### Interrogation of gene expression in human DLBCL cohorts

To assess prognostic relevance of gene expression in human DLBCL patients, the OSdlbcl web server was queried. A combination of 4 datasets were analyzed (TCGA, GSE21846, GSE32918 and GSE57611) generating a Kaplan-Meier (KM) curve stratifying patients by *IL2RB* using a 50% gene expression cutoff with log rank p value and hazard ratio (HR) reported as described^31^.

### Multiplex plasma cytokine assay and VEGFA ELISA

Plasma samples were thawed for multiplex cytokine analyses and vascular endothelial growth factor A (VEGFA) ELISA performed on the same day. Cytokine analyses were performed using a Milliplex MAP Magnetic Bead-based Canine Cytokine/Chemokine pre-mixed 13-plex kit (Millipore Sigma, Burlington, MA, USA, analyzed on MagPix Multiplexing System, Thermo Fisher Scientific, Waltham, MA, USA) according to the manufacturer’s instructions. Plasma VEGFA concentrations were determined using a canine VEGFA Quantikine ELISA Immunoassay (R&D Systems, Minneapolis, MN, USA), absorbances were read at 450 nm with 560 nm readings subtracted using a Glomax Discover Plate Reader (Promega Corporation, Madison, WI, USA). Concentrations were calculated by interpolation of the generated four parameter standard curve.

### Statistical analyses

Time to progression (TTP) was defined as the interval between initiation of CHOP and cytologically confirmed disease relapse/progression, patients were right censored if they were still in complete response at the time of transcriptional analyses. Median(m)TTPs were plotted using the Kaplan-Meier (KM) product-limit estimator and KM curves were compared using the log-rank test. For naive samples, groups were compared using two-tailed Mann-Whitney tests. Paired naive and relapsed samples were compared using two-tailed Wilcoxon matched-pairs signed rank test. Statistical significance was established at p < 0.05. Statistical analyses were performed using Prism v10 (GraphPad Software, Boston, MA, USA).

## Results

### Patient population

We recruited 18 dogs with intermediate-to-large BCL diagnosed by cytology of a peripheral nodal FNA for which PCR for antigen receptor rearrangement (PARR) and flow cytometry confirmed B cell neoplasia. Clonal rearrangements of the immunoglobulin heavy chain (IgH) were noted in all dogs, with one dog also having a concurrent T cell receptor (TCR) clonal rearrangement and another having a possible concurrent clonal rearrangement of the TCR. Flow cytometry was consistent with expansion of medium-sized CD21+ lymphocytes in all dogs, MHCII expression was high in 17 dogs and low in one, and CD34+ MHCII- precursor cells were not detected in any samples. There were 8 spayed females, 9 castrated males, and 1 intact male. At diagnosis, mean age was 8.1 years (range 3–12 years), and mean weight was 26.9 kg (range 7.3–52.0 kg). All 18 dogs had baseline CBCs, and 17 dogs had a baseline serum biochemistry panel. Baseline urinalysis was conducted in three patients with one other patient having urinalysis performed one month after diagnosis. Bone marrow sampling was not performed in any patient. Thoracic radiographs were performed in 11 patients, intrathoracic lymphadenopathy was noted in five patients, one additional patient had suspected pulmonary infiltration, another had suspected pulmonary infiltration and intrathoracic lymphadenopathy, and one dog had pleural effusion and lymphadenopathy. Abdominal ultrasound was performed in 12 patients, with suspected extension of lymphoma into the abdomen observed in all scanned patients, with infiltrated tissues including the liver, spleen and intraabdominal lymph nodes observed. One patient underwent echocardiography at diagnosis, and another underwent echocardiography prior to the first treatment with doxorubicin. Imaging findings were not confirmed by tissue sampling. Based on physical examination, blood results and diagnostic imaging, all dogs were at least World Health Organization (WHO) stage III, 14 dogs were substage a, and 4 dogs were substage b (Table 1)^32^. This cohort of patients all had comprehensive clinical follow-up and sufficient RNA isolated to perform NanoString profiling.

**Table 1:** Patient characteristics. *FS* female spayed, *MC* male castrated, *MI* male intact. *in remission at time of analyses, #failed to attain remission.

B cell patient number	Biobank sample number(s)	Age	Sex	Breed	Weight	Minimum Stage	Substage	PARR	Flow cytometry	TTP
1	Naive: 1 Relapse: N/A	12	MC	Mixed	16.8	4	a	IgH clonal	CD21 lymphocytosis MHCII high	1002*
2	Naive: 3 Relapse: 15	4	MI	Mixed	12.5	4	a	IgH clonal	CD21 lymphocytosis MHCII low	245
3	Naive: 4 Relapse: 5	7	FS	Mixed	11.8	5	a	IgH clonal	CD21 lymphocytosis MHCII high	183
4	Naive: 6 Relapse: 7	10	MC	Mixed	22.6	5	a	IgH clonal	CD21 lymphocytosis MHCII high	133
5	Naive: 8 Relapse: N/A	9	MC	Mixed	7.3	5	a	IgH clonal and TCR possibly clonal	CD21 lymphocytosis MHCII high	883*
6	Naive: 12 Relapse: 56	10	FS	Mixed	28.0	3	a	IgH clonal	CD21 lymphocytosis MHCII high	274
7	Naive: 24 Relapse: N/A	8	MC	Australian Cattle Dog	31.8	5	a	IgH clonal	CD21 lymphocytosis MHCII high	819*
8	Naive: 25 Relapse: 62	8	MC	Mixed	28.9	4	b	IgH clonal	CD21 lymphocytosis MHCII high	251
9	Naive: 26 Relapse: 63	3	FS	English Bulldog	28.2	5	b	IgH clonal	CD21 lymphocytosis MHCII high	467
10	Naive: 27 Relapse: 64	10	MC	Mixed	33.0	4	a	IgH clonal	CD21 lymphocytosis MHCII high	434
11	Naive: 29 Relapse: N/A	8	MC	Labrador	40.5	3	a	IgH clonal	CD21 lymphocytosis MHCII high	783*
12	Naive: 30 Relapse: N/A	8	FS	Mixed	30.1	4	a	IgH clonal	CD21 lymphocytosis MHCII high	741*
13	Naive: 31 Relapse: 65	10	MC	Samoyed	36.0	4	b	IgH clonal	CD21 lymphocytosis MHCII high	313
14	Naive: 32 Relapse: N/A	6	FS	Boerboel	52.0	5	a	IgH clonal and TCR clonal	CD21 lymphocytosis MHCII high	8#
15	Naive: 37 Relapse: 66	10	FS	Mixed	28.5	3	a	IgH clonal	CD21 lymphocytosis MHCII high	127
16	Naive: 45 Relapse: N/A	12	FS	Mixed	29.0	5	b	IgH clonal	CD21 lymphocytosis MHCII high	43#
17	Naive: 57 Relapse: 58	7	MC	Mixed	8.1	3	a	IgH clonal	CD21 lymphocytosis MHCII high	239
18	Naive: 59 Relapse: 60	5	FS	Rottweiler	39.8	3	a	IgH clonal	CD21 lymphocytosis MHCII high	230

### Treatment outcomes

**Fig. 2:**
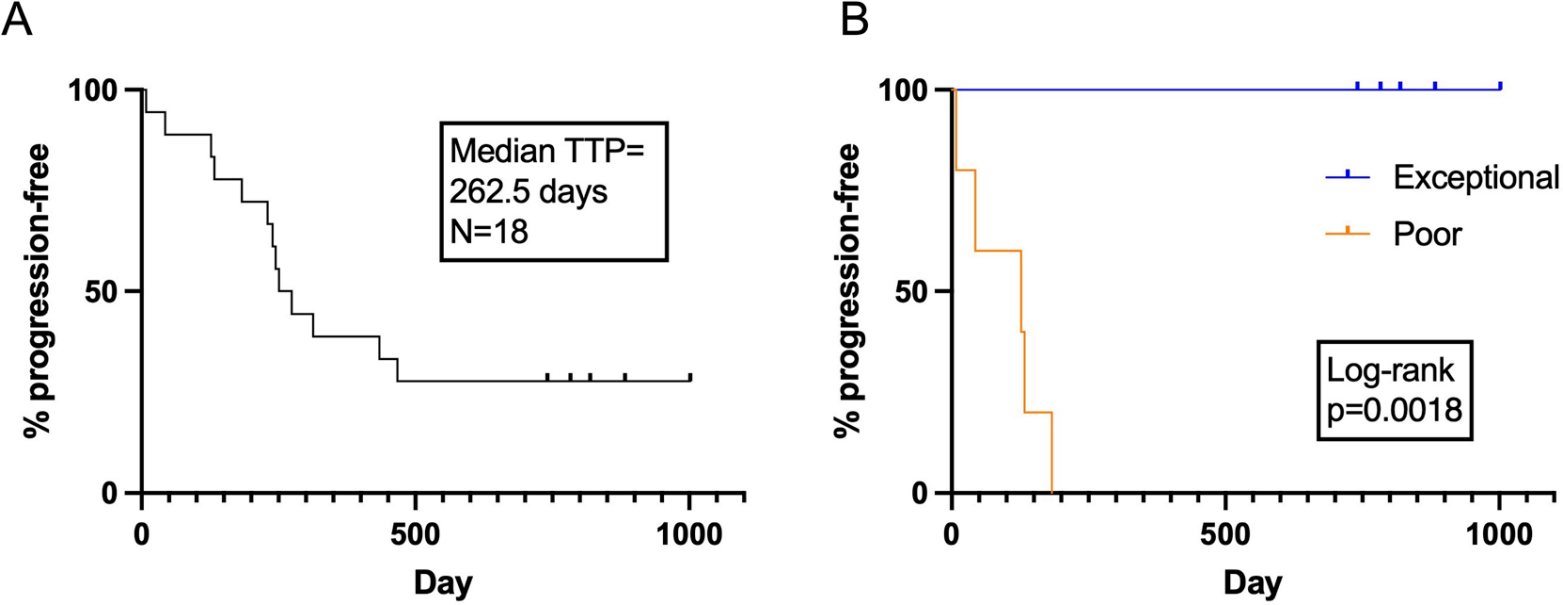
Time to progression for dogs diagnosed with aggressive BCL. Kaplan-Meier curves for (**A**) all 18 enrolled dogs and (**B**) five exceptional and five poor responders. Curves compared using log-rank test.

First-line therapy was initiated with CHOP and disease burden was monitored throughout treatment and at routine follow-up after CHOP. When clinically suspected based on lymph node size, disease progression was confirmed by cytology in all cases. The mTTP was 262.5 days (range 1-1002 days) for all dogs (Fig. 2a). Thirteen dogs completed the course of CHOP. One patient had vinblastine substituted after 3 doses of vincristine prior to receiving single-agent doxorubicin as a result of ileus (patient 5) and thus did not complete CHOP, and four other patients progressed prior to completing CHOP (patients 4, 14, 15 and 16). One dog received L-asparaginase prior to CHOP (patient 9). Two dogs (patients 3 and 18) received seven of the eight planned doses of vincristine with one dog (patient 12) receiving six of eight doses at the attending clinician’s discretion. One dog had two doses of vincristine substituted for vinblastine due to a mast cell tumor being diagnosed in the final round of CHOP. At the time of data analyses, five dogs were progression free (exceptional responders) and had significantly longer TTP than the five worst responders (poor responders) that included two dogs that failed to achieve remission following CHOP (Fig. 2b). Taken together, this generated a minimally invasive biobank for dogs with aggressive BCL with linked hematologic, cytologic, immunophenotypic, and clinical metadata enabling us to interrogate factors associated with disease outcome in this cohort.

### Transcriptional profiling of canine BCL patients

To interrogate the immune TME, NanoString transcriptional profiling was performed on naive samples from all 18 dogs, which included the five exceptional responders that had not relapsed and the two dogs that failed to gain remission after initiating CHOP. A subset of 11 patients had paired naive samples pre-CHOP with subsequent samples taken at time of relapse (Table 1).

**Fig. 3:**
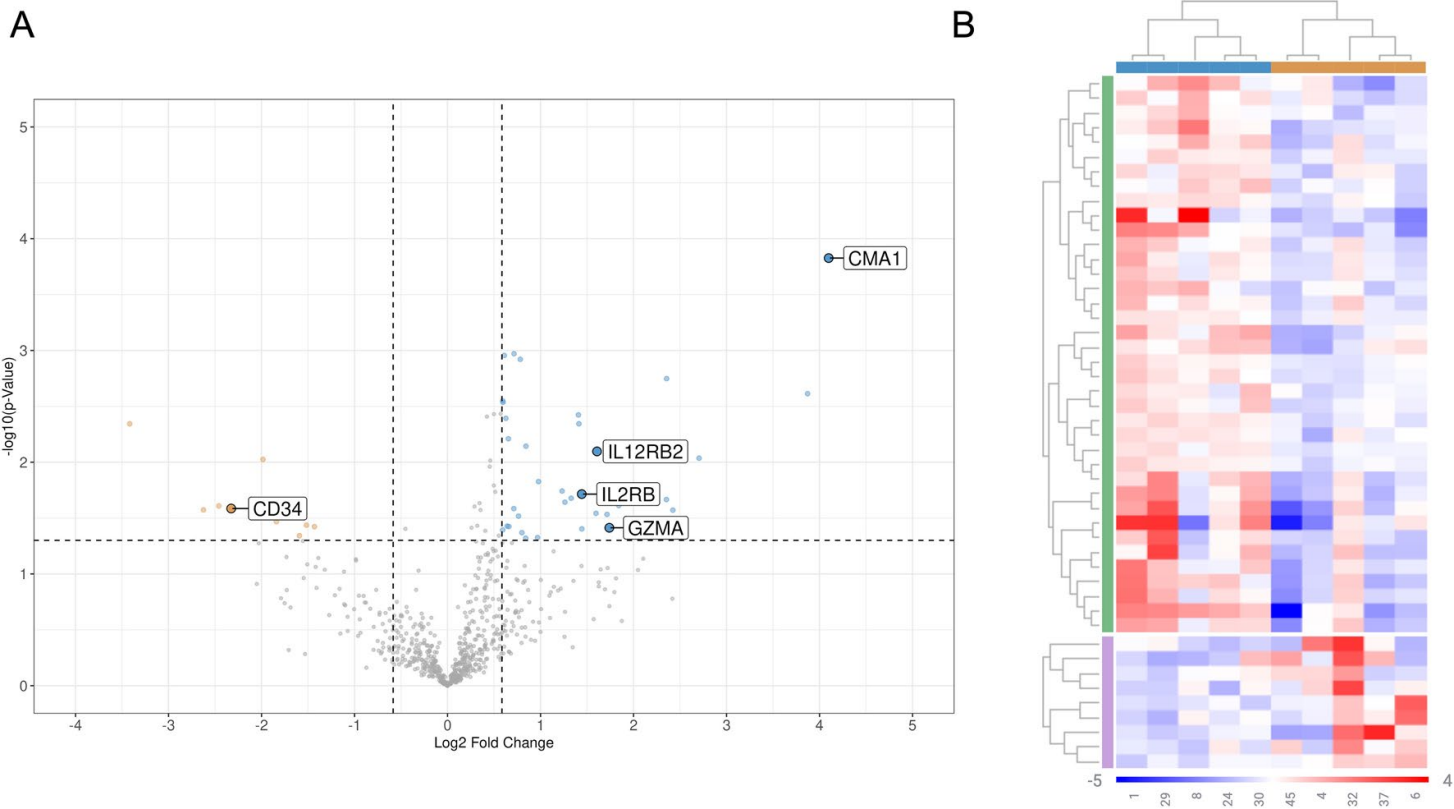
Transcriptional profiling of exceptional and poor canine BCL patients. (**A**) volcano plot and (**B**) gene clustering depicted by heatmap. Data derived from NanoString canine IO panel and analyzed using the ROSALIND platform, colors of the heatmap represent log2 normalized gene expression after subtracting the mean on a per-gene basis, blue dots on volcano plot corresponds to blue horizontal bar at the top of heat map and depict genes enriched in exceptional responders, orange dots on volcano plot corresponds to orange horizontal bar at the top of heat map and depicts genes enriched in poor responders.

**Fig. 4:**
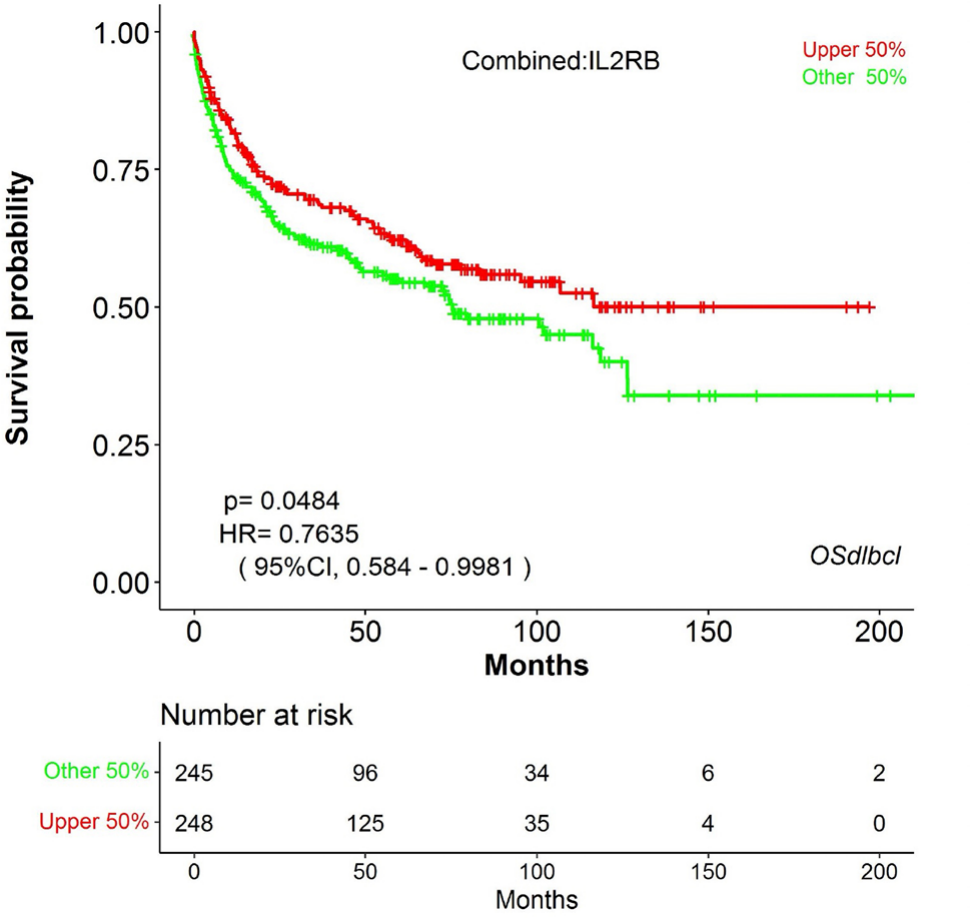
Survival times of people diagnosed with DLBCL stratifying patients by *IL2RB* using a 50% gene expression cutoff. The OSdlbcl web server was queried and a combination of 4 datasets were analyzed (TCGA, GSE21846, GSE32918 and GSE57611) generating a Kaplan-Meier (KM) curve with log rank p value and hazard ratio (HR) displayed.

For initial naive profiling, stratification of the 18 dogs above and below the mTTP revealed seven differentially expressed genes (DEGs) that were enriched in dogs maintaining remission beyond the mTTP and two genes that were enriched in dogs with remissions below the mTTP (Fig. S1a and Table S2). No clear segregation of patients based on DEG clustering was noted when stratifying all patients by mTTP (Fig. S1b). To discern potential DEGs associated with exceptional and poor outcomes, we compared the transcriptional profiles of the five exceptional to the five worst responders. This revealed 38 DEGs enriched in exceptional responders, including multiple genes associated with T cell function and homing alongside 9 DEGs enriched in the poor responders (Fig. 3a; Table 2). Segregation of exceptional and poor responders was noted in non-biased clustering of gene expression profiles (Fig. 3b). In both naive analyses, *CMA1*,* GZMA*,* IL12RB2* and *IL2RB* were enriched in dogs with longer remissions and *CD34* was enriched in dogs with shorter remissions (Figs. S1a and 3a, Table 2 and S2). As increased *IL2RB* was associated with increased survival in an independent study of canine DLBCL patients undergoing chemo-immunotherapy, we investigated the translational relevance of this gene in human patients and found increased *IL2RB* was also associated with prolonged overall survival in a combination of 4 human DLBCL studies (Fig. 4)^25^.

**Table 2:** Differentially expressed genes for exceptional vs. poor responders.

Name	Fold change	p-value	Name	Fold change	p-value
Enriched in exceptional responders					
*CMA1*	17.125	0.000149	*IL2RB*	2.71948	0.01929
*CD79A*	1.64142	0.001068	*NCF4*	2.51218	0.020996
*PSMB10*	1.52663	0.001108	*FGFR3*	5.11112	0.02158
*LY86*	1.71998	0.001199	*AMICA1*	2.39888	0.022781
*FCRL2*	5.11796	0.001786	*GZMK*	3.58186	0.024446
*CCR4*	14.6337	0.002437	*SHMT2*	1.6385	0.026014
*LAP3*	1.50813	0.00282	*CXCL10*	5.36278	0.026833
*MYD88*	1.51239	0.002911	*KLRA1*	3.023	0.028654
*IFGGC1*	2.65194	0.003771	*LCN2*	3.2826	0.029304
*KLRG1*	1.5446	0.004049	*TNFSF14*	1.69714	0.030351
*ANXA1*	2.66204	0.004528	*CD3D*	1.55896	0.037327
*CD37*	1.57395	0.00617	*LAG3*	1.57988	0.037641
*GBP5*	1.79377	0.007181	*GZMA*	3.33987	0.038609
*NKG7*	3.29017	0.007847	*KLRK1*	2.72085	0.03948
*IL12RB2*	3.04652	0.008004	*FAM26F*	1.50821	0.040373
*LOC490356*	3.61571	0.008918	*XCR1*	4.06251	0.042627
*CPA3*	6.51723	0.009201	*IL15RA*	1.74074	0.042704
*CD247*	1.96905	0.01492	*LOC100049001*	1.95604	0.047233
*KLRB1*	2.34832	0.018107	*TNFSF10*	1.79087	0.047838
Enriched in poor responders					
*CCL25*	-10.6875	0.004531			
*MST1R*	-3.95832	0.009448			
*SLC16A3*	-5.49999	0.02462			
*CD34*	-5.01556	0.025966			
*PDPN*	-6.16666	0.026719			
*EGR1*	-3.58147	0.03403			
*CDKN2A*	-2.86183	0.036571			
*CXCR4*	-2.69667	0.037747			
*PTK2*	-3.01532	0.045458			

We next compared how transcriptomic profiles were influenced by time of sampling using paired naive and relapsed samples and found 26 DEGs enriched in naive aspirates and 29 DEGs enriched at relapse (Figure S2a and Table S3). Monocytic and cytokine transcripts including *CD163*,* IFNB1*,* IL10*, and *TGFB2* were enriched in naive samples. At relapse, various transcripts involved in T cell activity, including *IL2RB*, were enriched. Interestingly, *VIM* (encoding for vimentin) was the most significantly enriched DEG and the T cell checkpoint *CD276* was also enriched at relapse. Clustering of gene signatures was not clearly segregated by the time point at which the sample was acquired, although a subset of four dogs (dogs 6, 8, 9, and 17) clustered together based primarily on upregulation of T cell transcripts at relapse (Figure S2b). Collectively, we revealed transcriptional differences in innate and adaptive immune components associated with survival and disease progression.

### Screening for circulatory biomarkers in canine BCL patients

**Fig. 5:**
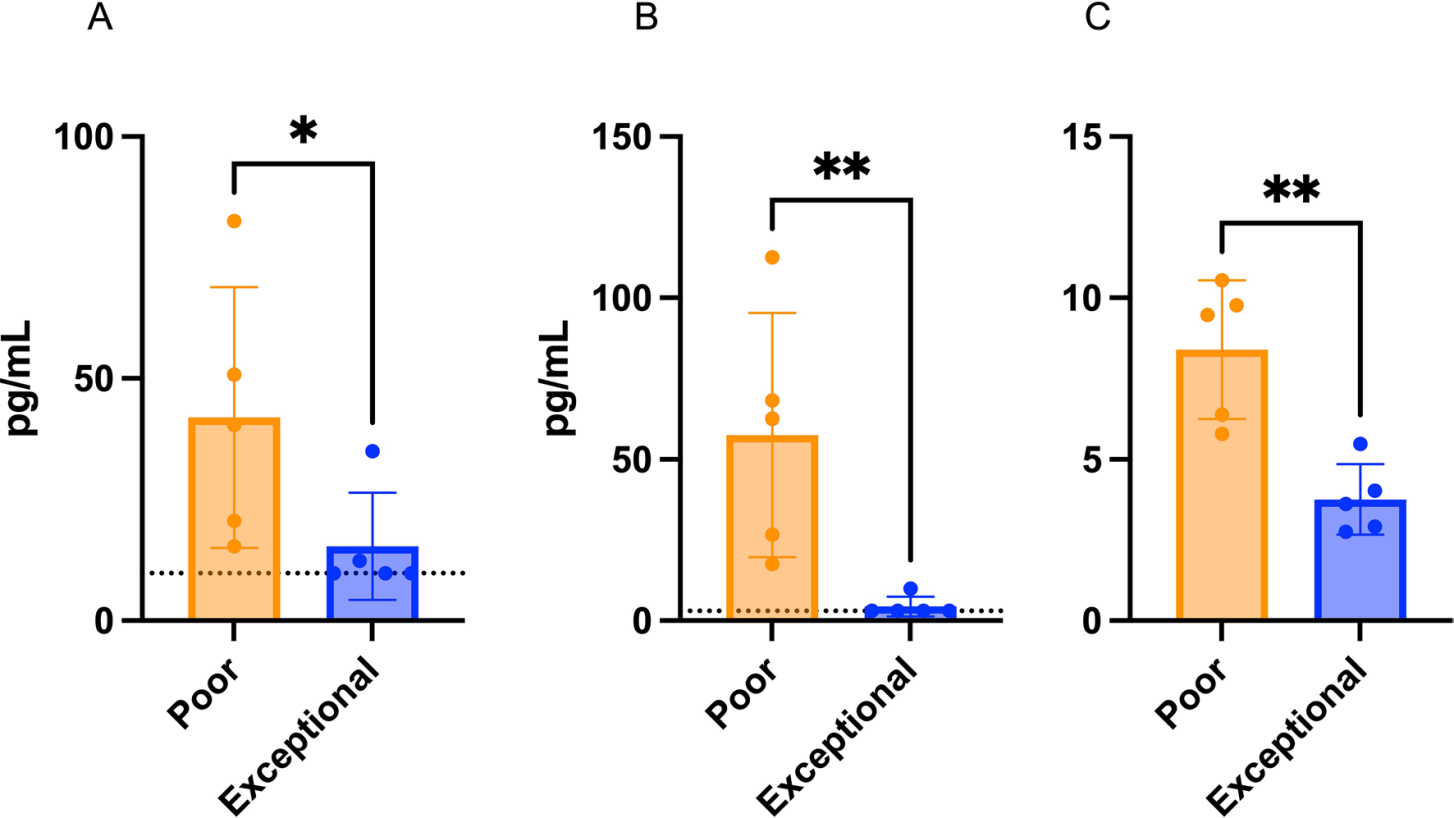
Circulating biomarker profiling for aggressive canine BCL in five dogs with exceptional responses and five dogs with poor responses to CHOP chemotherapy. (**A**) plasma VEGFA concentrations, (**B**) plasma IL-6 concentrations, and (**C**) neutrophil-to-lymphocyte ratio. Comparisons between two groups performed using two-tailed Mann-Whitney tests, p< *0.05, **0.01. Dotted lines represent limit of detection.

To screen for circulatory biomarkers, we analyzed cryopreserved plasma samples. Although *CD34* expression was increased in dogs with shorter TTPs, diagnostic flow cytometry failed to detect the presence of CD34 + precursor B cells in any of our cases. As circulating proangiogenic CD34 + hematopoietic stem and progenitor cells have been identified in dogs with various malignancies, we sought to further investigate potential markers of angiogenesis in the circulation by performing plasma VEGFA ELISAs^33^. When comparing all naive samples stratified by the mTTP, we found a non-significant trend of higher plasma VEGFA at baseline in dogs with shorter TTP (Figure S3a). However, a significantly higher VEGFA concentration in the poor responders compared to the exceptional responders was documented (Fig. 5a). Plasma VEGFA was also significantly higher in treatment naive samples compared to relapse (Figure S4a).

To further characterize systemic immunity in our patients, we performed multiplex cytokine and chemokine profiling alongside analyses of circulating white cell counts determined by baseline CBCs. Plasma IL-6 levels were significantly higher in dogs with shorter TTPs when stratifying all dogs by mTTP and when comparing poor and exceptional responders (Figures S3b and 5b). A significantly higher circulating neutrophil-to-lymphocyte ratio (NLR) was also found in dogs with shorter remissions in both groups (Figures S3c and 5c).

Multiplex plasma cytokine profiling revealed additional differences between naive and relapsed samples with significantly higher concentrations of KC-like (Figure S4b), IL-10 (Figure S4c) and MCP-1 (Figure S4d) recorded in naive samples. Thus, we detected changes in systemic immunity that were associated with patient outcomes, and time of sample acquisition, that likely reflected both tumor- and chemotherapeutic-induced changes.

## Discussion

In this prospective study of canine BCL patients undergoing multi-agent chemotherapy, we were able to document changes in immunity associated with remission durations and time of sampling within the immune TME and peripheral circulation. We found that multiple T cell transcripts were enriched in patients with prolonged remissions, whereas markers associated with pro-angiogenesis and activation of the innate immune system were associated with short remission. With regards to timing of sampling, T cell transcripts were enriched within a subset of relapsed samples, with markers associated with pro-angiogenesis and innate immunity enriched in treatment naive samples. Collectively, these findings provide evidence that the pre-treatment immune status of dogs diagnosed with BCL impacts outcomes of chemotherapy and that immune responses evolve over time following cytotoxic chemotherapy, in line with findings in human DLBCL.

Amongst other T cell associated transcripts, *GZMA*,* IL12RB2*, and *IL2RB* were enriched in exceptional responders and dogs maintaining remissions above the mTTP. Granzyme A (*GZMA*) is typically stored within the cytotoxic granules of T and natural killer (NK) cells and, following immune synapse formation, exerts cytotoxicity by targeting the nucleus of neoplastic or infected cells^34^. IL-12 induces anti-tumor mediated immunity via signaling through the IL-12 receptor (IL-12R) which is found on various effector cells including T and NK cells^35^. The IL-12R is composed of two subunits- IL12Rβ1 and IL12Rβ2 (*IL12RB2*), with the latter only present on T cells following activation^35^. Notably, *Il12rb2* knockout mice develop an autoimmune and lymphoproliferative disorder characterized by oligoclonal B cell proliferation and up-regulation of systemic IL-6^36^, suggesting that increased IL12 signaling mediates anti-BCL immunity. IL-2 signals through the IL-2 receptor (IL-2R) which is comprised of either a high affinity heterotrimer or intermediate affinity heterodimer, both of which contain the IL-2Rβ subunit (*IL2RB*)^37^. As IL-2R is found on a variety of immune effector and regulatory cell types, including effector T cells, regulatory T cells (Tregs), and NK cells, signaling through IL-2 can have pleiotropic effects upon anti-tumor immunity. However, clinical responses have been noted in DLBCL patients following high dose IL-2 treatment^37,38^. Work by Licenziato et al. revealed a gene signature that included enrichment of *IL2RB* that was associated with positive outcomes in canine lymphoma patients undergoing chemo-immunotherapy. Our study supports this finding and further revealed that *IL2RB* enrichment is favorable in human DLBCL patients. Whilst further investigations of the functional implications of our transcriptomic findings are warranted, collectively our finding of increased T cell transcripts is aligned with literature whereby greater T cell numbers in the TME of human DLBCL are associated with favorable patient outcomes^39,40^. Indeed, redirecting T cell immunity with chimeric antigen receptor T cell therapy has transformed the treatment of human DLBCL patients. However, as many human patients do not respond, next-generation CAR T approaches are needed, and our findings suggest that canine BCL could provide a powerful setting to streamline novel immunotherapies into the human clinic^4^.

Increased NLR, a recognized biomarker for inflammation, was identified as a negative prognostic indicator in a meta-analysis of 2515 human DLBCL patients^41^. Increased NLR was also associated with poor outcomes in a separate study of canine DLBCL patients undergoing CHOP chemotherapy^42^. In human DLBCL, the presence of CD34+ endothelial cells were found to be consistent with induction of angiogenesis within the TME and angiogenic signatures are associated with poor treatment outcomes^43–46^. Our findings revealed that *CD34* transcripts were enriched in dogs with shorter remissions alongside increased circulatory VEGFA concentrations, consistent with previous studies showing high serum VEGFA was associated with poor outcomes in various subtypes of human and canine lymphoma^47,48^. Increased circulating IL-6 and NLR were also observed in our poor responders. Broadly, IL-6 favors tumor progression and amongst other functions, promotes angiogenesis and suppresses anti-tumor immunity^49^. Increased circulatory IL-6 has been long established as a negative prognostic indicator in human lymphoma patients and simultaneous elevations of IL-6 and VEGFA are independent predictors of survival in aggressive NHL^50,51^. Interestingly *CMA1*, encoding for mast cell chymase, was the most enriched gene in dogs with prolonged remissions. This is consistent with findings in human DLBCL where increased intratumoral mast cells confer favorable outcomes^52^. Paradoxically, mast cells are associated with increased angiogenesis in human DLBCL, underscoring the need to further study their role in DLBCL^53,54^. To date, targeting angiogenesis has had limited success at improving outcomes in DLBCL, likely due to signaling redundancy and undesirable toxicity profiles. However, novel treatment concepts, including vascular normalization strategies should be considered to improve anti-BCL therapies^55^.

When comparing naive with relapsed samples, we also observed changes in immune compositions within the TME and systemically. Locally, several genes encoding for cytokines were enriched in naive samples including *IFNB1*,* TGFB2*, and *IL10* alongside *CD163*, with the latter a marker of M2 macrophages^56^. In human DLBCL, *CD163* is upregulated in intratumoral macrophages^57^. We also documented higher circulating VEGFA, IL-10, MCP-1, and KC-like concentrations in naive samples. As macrophages act as a significant source for many of these cytokines, further investigation into the role of macrophages and how these cellular populations change following treatment of canine lymphoma is indicated^56^. Vimentin (encoded by *VIM*) was the most significantly upregulated gene at relapse in dogs and increased vimentin has been associated with a CHOP resistant phenotype in human DLBCL cells implicating vimentin as a putative factor mediating invasive, multi-drug resistant disease^58^. Notably, T cell transcripts, including *IL2RB*, were enriched at relapse and were primarily driven by a subset of patients. There was no differential expression of granzyme encoding genes in this setting, however, we did note enrichment of *CD276* encoding for the T cell checkpoint B7-H3 which exhibits increased expression at the mRNA level in human DLBCL^59,60^. Further studies into changes of functionality of T cell subsets and potential shifts from granzyme associated cytotoxicity to T cells impeded by overexpression of checkpoints such as B7-H3 are indicated. As dog owners are often not trained in the palpation of lymph nodes and BCL is not always accompanied by systemic signs of illness, neoplastic nodes may be markedly enlarged at diagnosis^15,16^. This is somewhat contrary to the situation of frequent surveillance of nodes performed by veterinary oncologists during and after CHOP treatment, where relapse may be identified following minor increases in nodal diameter. Thus, whilst our findings justify future studies, our data should be taken in the context of potentially differing tumor burdens between naive and relapsed timepoints as we speculate that disease burden at diagnosis may be greater than that at relapse and thus may impact immune contexture.

Whilst our study was prospective, some limitations were identified. As FNAs provide a minimally invasive, cost effective, and rapid methodology to diagnose aggressive canine BCL, they are widely relied upon by veterinary oncologists in the clinical workup of cases^61^. However, to obtain full WHO classification of lymphoma, surgical removal of a node with subsequent histologic and immunohistochemical examinations are required^17^. A further limitation was the lack of complete staging for all patients which may have resulted in underestimating the WHO stage in some dogs. Finally, the numbers of patients in our analyses were small. The general paucity of prior data on the influence of the immune profiling techniques employed here on the outcomes of canine BCL patients receiving CHOP precluded us from predicting a potential effect size and thus performing a reliable power analysis when designing our study. However, the data generated from our current pilot study can now be used to ensure subsequent prospective studies are adequately powered.

Beyond identifying putative biomarkers in canine BCL patients, our data further supports the fidelity of studying pet dogs as a parallel patient population to human DLBCL. Our approach enabled the generation of data utilizing a minimally invasive technique, and these findings will guide subsequent targeted investigations of specific immune and stromal cell subsets within the TME of canine BCL. Future investigations utilizing spatial profiling in combination with standard histologic exams of extirpated lymph nodes alongside single cell transcriptomic and multi-parameter flow cytometry are planned to comprehensively characterize the immune profile in canine BCL. Such work may further contribute to the rationale of performing future targeted interventional studies in canine patients to inform the design of early phase human trials^13^.

## Supplementary Information

Below is the link to the electronic supplementary material.

Supplementary Material 1

## Data Availability

All data is available from the corresponding author upon reasonable request.
