# Supplementary material for: Immune profiling of canine B cell lymphoma reveals cross-species conservation of prognostic markers
Source: Sci Rep. 2025 Aug 4;15:28385. doi: 10.1038/s41598-025-13389-2 (PMC12322273; doi:10.1038/s41598-025-13389-2)
Supplement: Supplementary file 1 — Supplementary Material 1 [file 41598_2025_13389_MOESM1_ESM.pdf]

Supplementary data

Figure S1 Transcriptional profiling of canine B cell lymphoma patients stratified by median time to progression (mTTP). **A.** volcano plot and **B.** gene clustering depicted by heatmap (Data derived from NanoString canine IO panel and analyzed using the ROSALIND platform, colors of the heatmap represent log2 normalized gene expression after subtracting the mean on a per-gene basis, blue dots on volcano plot corresponds to blue horizontal bar at the top of heat map and depict genes enriched in dogs with responses above mTTP, orange dots on volcano plot corresponds to orange horizontal bar at the top of heat map and depicts genes enriched in dogs with responses below mTTP).

Figure S2 Transcriptional profiling of canine B cell lymphoma patients comparing naive and relapsed samples. **A.** volcano plot and **B.** gene clustering depicted by heatmap (Data derived from NanoString canine IO panel and analyzed using the ROSALIND platform, colors of the heatmap represent log2 normalized gene expression after subtracting the mean on a per-gene basis, blue dots on volcano plot corresponds to blue horizontal bar at the top of heat map and depict genes enriched in relapses samples, orange dots on volcano plot corresponds to orange horizontal bar at the top of heat map and depicts genes enriched in naive samples).

Figure S3 Circulating biomarker profiling for aggressive canine B cell lymphoma patients stratified by median time to progression (mTTP). **A.** plasma VEGFA concentrations, **B.** plasma IL-6 concentrations, and **C.** neutrophil-to-lymphocyte ratio. (Comparisons between two groups

performed using two-tailed Mann-Whitney tests, p< *0.05, **0.01, ns = not significant. Dotted lines represent limit of detection).

Figure S4 Circulating biomarker profiling for aggressive canine B cell lymphoma patients comparing naive and relapsed samples. **A.** plasma VEGFA concentrations, **B.** plasma KC-like concentrations, **C.** plasma IL-10, and **D.** plasmaMCP-1. (Comparisons between two groups performed using two-tailed paired Wilcoxon tests, p< *0.05, **0.01. Dotted lines represent limit of detection).

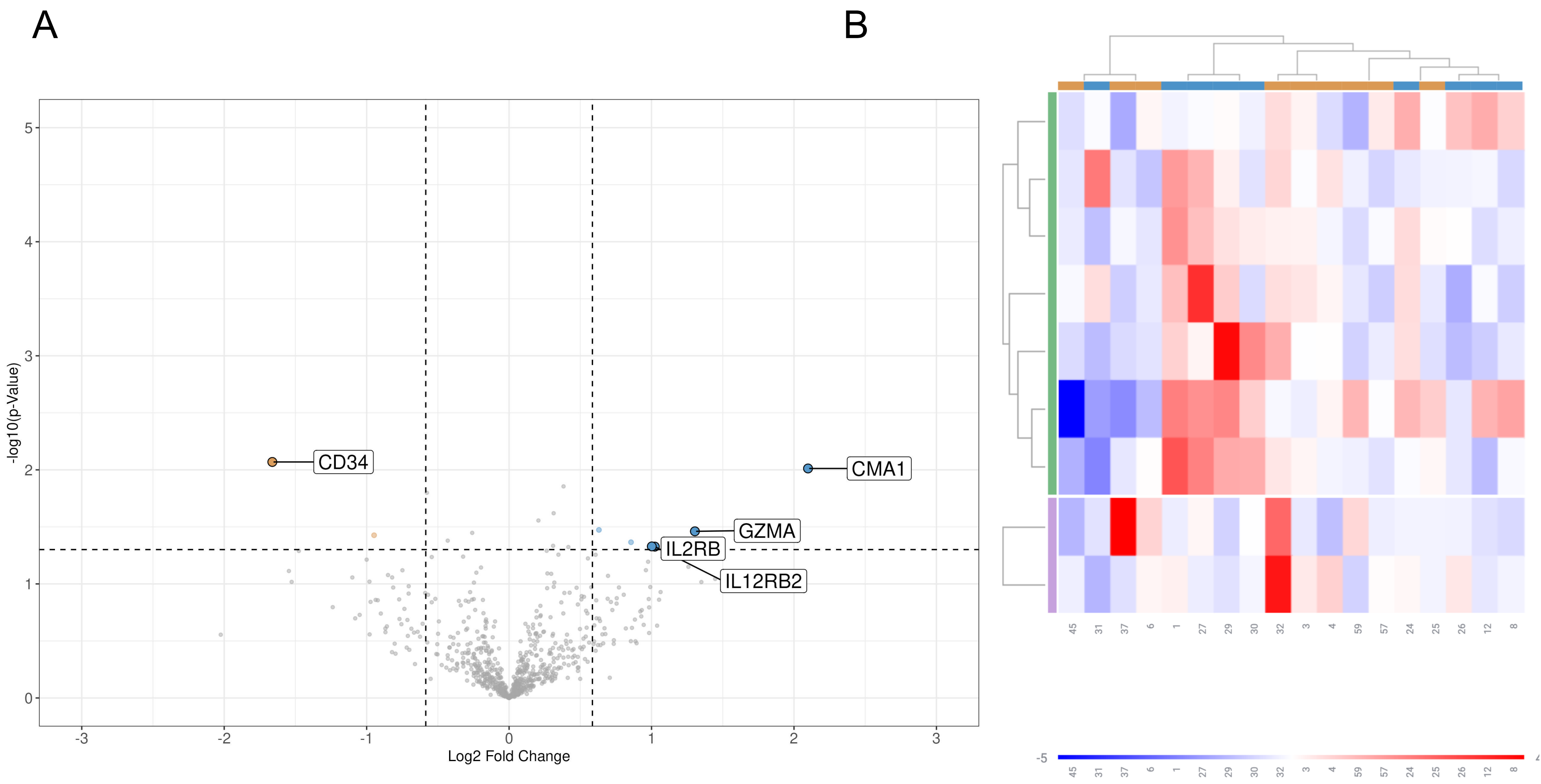
A
B
CD34
CMA1
GZMA
IL2RB
IL12RB2
-log10(p-Value)
Log2 Fold Change
-3
-2
-1
0
1
2
3
4
5
45
31
37
6
1
27
29
30
32
3
4
59
57
24
25
26
12
8
-5

Figure S1

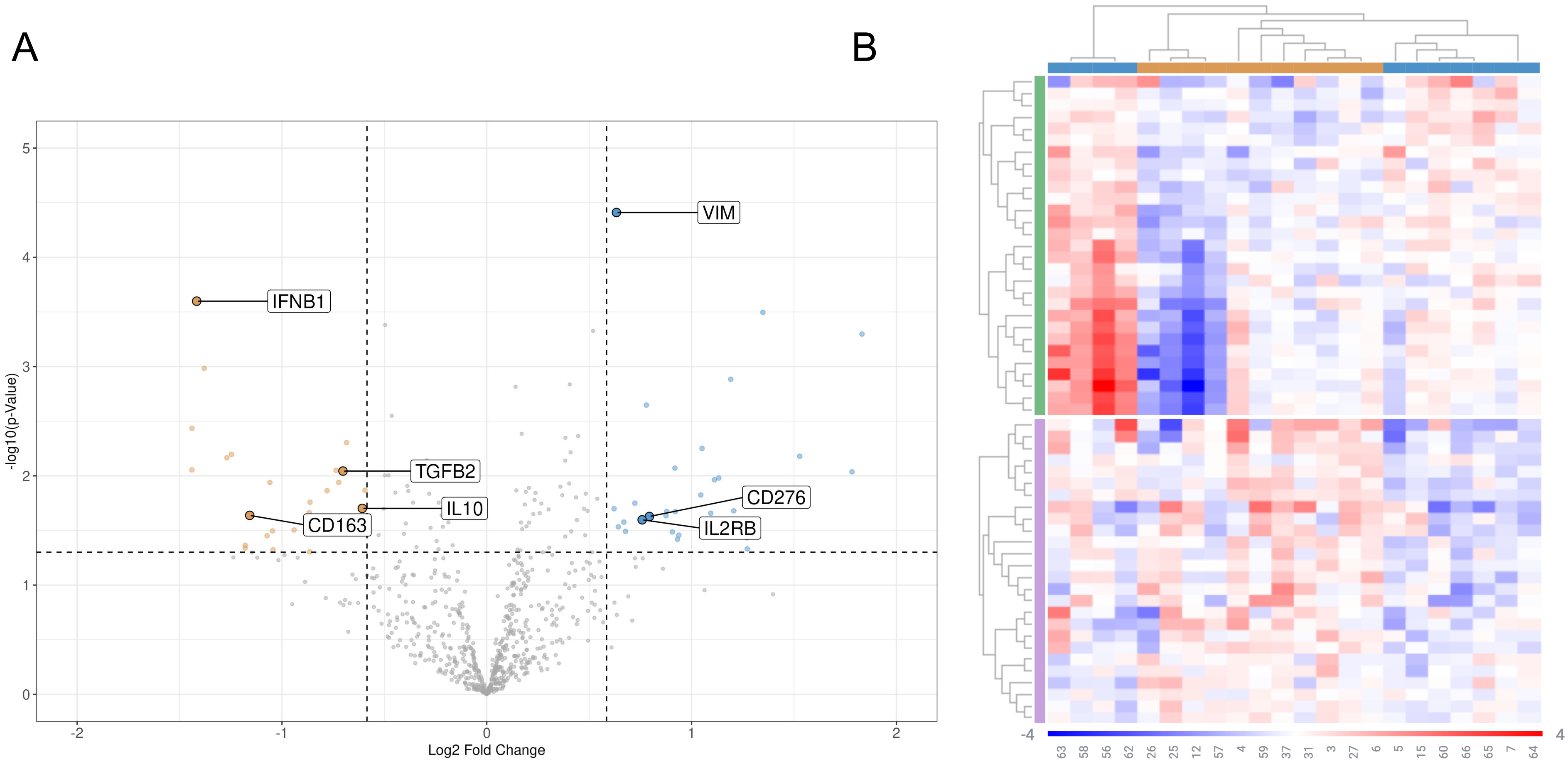
A
B
IFNB1
VIM
TGFB2
IL10
CD163
CD276
IL2RB
-log10(p-Value)
Log2 Fold Change
-2
-1
0
1
2
5
4
3
2
1
0
-4
4
63
58
56
62
26
25
12
57
4
59
37
31
3
27
6
5
15
60
66
65
7
64

Figure S2

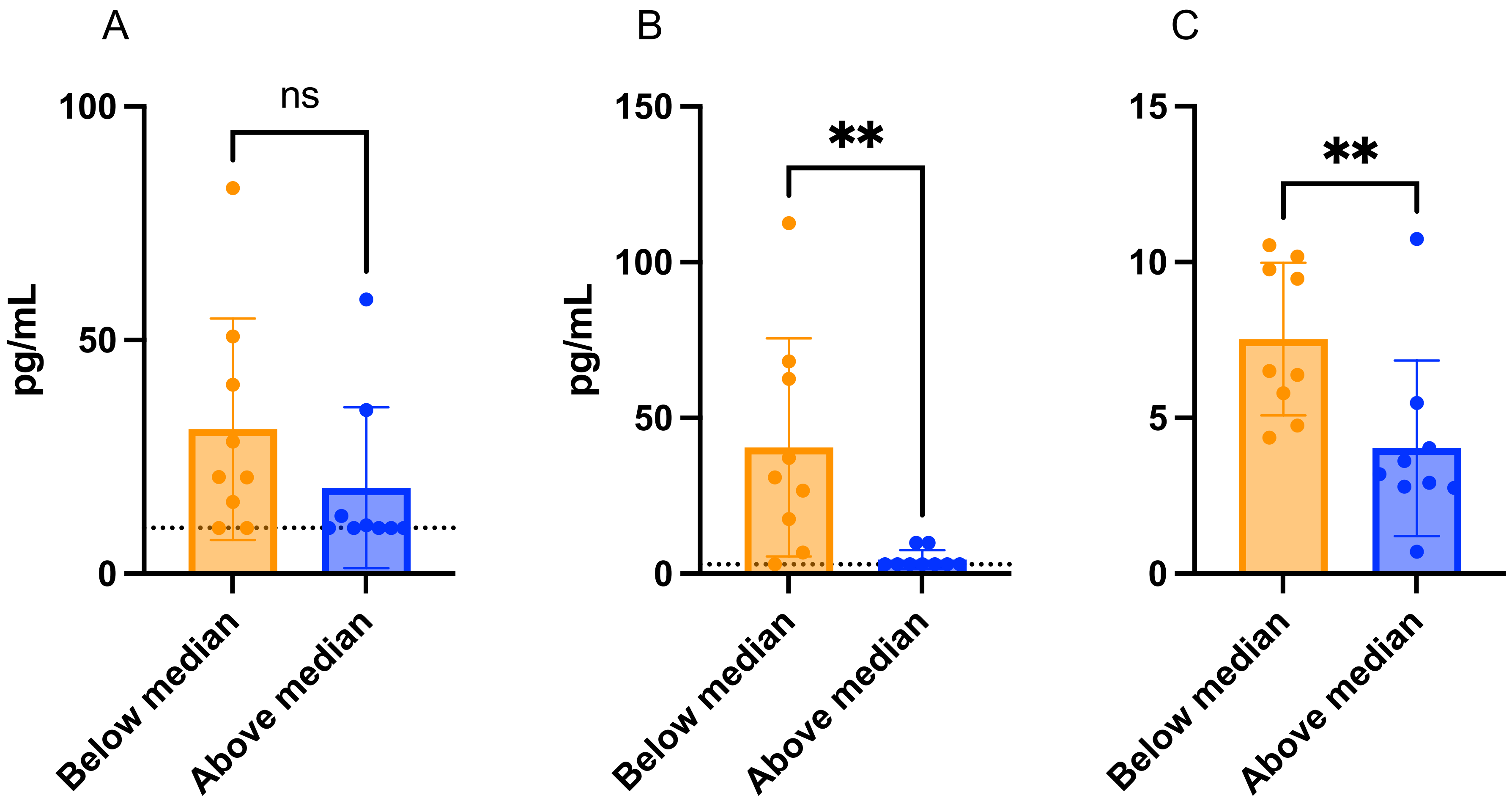
A
B
C
ns
**
**
pg/mL
pg/mL
0
50
100
0
50
100
150
0
5
10
15
Below median
Above median
Below median
Above median
Below median
Above median

Figure S3

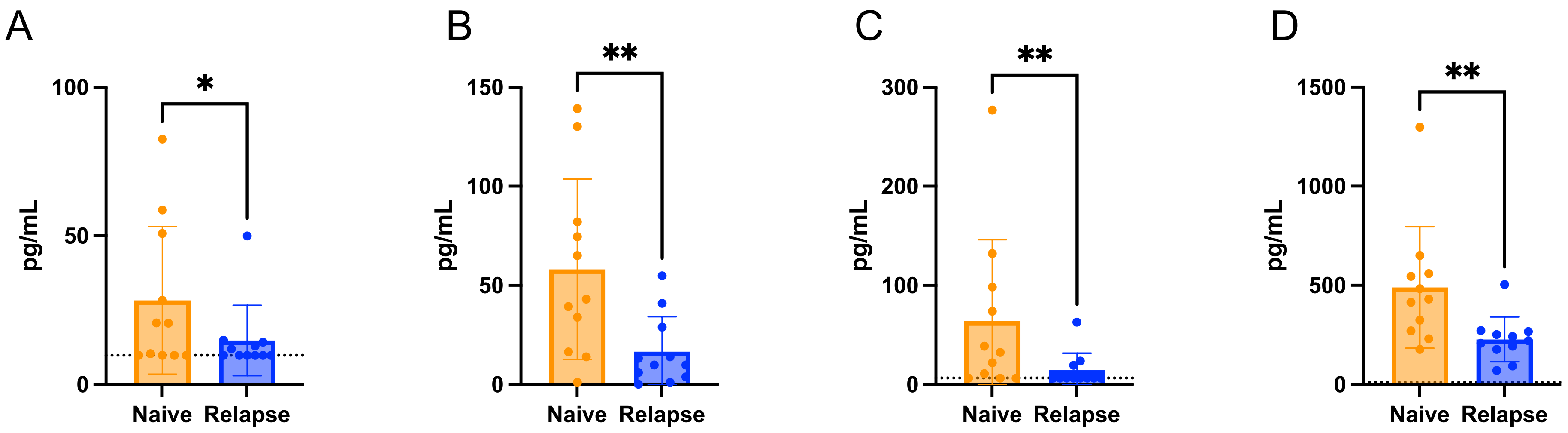

Figure S4

Table S1 CHOP protocol (IV intravenous, PO per os)

Drug	Week																			
	**1**	**2**	**3**	**4**	**5**	**6**	**7**	**8**	**9**	**10**	**11**	**12**	**13**	**14**	**15**	**16**	**17**	**18**	**19**	**20**
**Vincristine**<br>0.6-0.7 mg/m^2 IV<br>(<15 kg= 0.5 mg/m^2)	•<br>0.6		•<br>0.7			•		•			•		•			•		•		
**Cyclophosphamide**<br>250-300 mg/m^2 PO<br>+ 2 mg/kg PO Furosemide		•<br>250					•<br>275					•<br>300					•			
**Doxorubicin**<br>30 mg/m^2 IV<br>(<15 kg=1 mg/kg)				•					•					•					•	
**Prednisone**<br>40 mg/m^2 PO taper	•	•	•	•																

Dosages for vincristine and cyclophosphamide were increased as indicated depending on tolerability, nadir neutrophil and platelet count, and clinician discretion. Patients weighing less than 15 kg were dosed at 1 mg/kg doxorubicin.

Table S2 Differentially expressed genes stratified by median time to progression (mTTP)

Name	Fold Change	p-Value	Name	Fold Change	p-Value
**Enriched in patients above mTTP**			**Enriched in patients below mTTP**		
CMA1	4.28261	0.009724	***CD34***	-3.16486	0.008528
IL7	1.54818	0.033622	***CCND3***	-1.9289	0.037438
GZMA	2.46972	0.034587			
LGALS3	1.80992	0.043102			
IL2RB	2.00266	0.046912			
IL12RB2	2.03225	0.047104			
CD9	2.22109	0.048695			

Table S3 Differentially expressed genes between naive and relapsed samples

Name	Fold Change	p-Value	Name	Fold Change	p-Value
**Enriched in relapsed**					
VIM	1.55081	0.000039	***CXCR3***	1.83829	0.021245
MAPK11	2.5455	0.000319	***EGR1***	1.89235	0.021278
CX3CR1	3.55974	0.000504	***ITK***	2.13407	0.021991
DDR1	2.28319	0.001307	***IGHG***	1.83408	0.02318
AMICA1	1.71604	0.002253	***CD276***	1.73383	0.023557
NINJ2	2.07174	0.005609	***IL2RB***	1.69183	0.025267
CD6	2.8832	0.006625	***ITGA1***	1.59033	0.026531
CD8A	1.88999	0.008498	***RORA***	1.56051	0.029326
TRGC3	3.44177	0.009186	***IL26***	1.59925	0.032257
TRAT1	2.19258	0.010475	***TRBC***	1.87399	0.032621
CD5	2.16021	0.010868	***ARG2***	1.91518	0.034918
CD8B	2.06314	0.014988	***CCL17***	2.41193	0.03688
PVR	1.65045	0.017781	***TRAC***	1.90633	0.038085
TNFRSF17	1.53826	0.020008	***CD28***	2.41441	0.046635
TCF7	2.3067	0.020923			
**Enriched in naive**					
IFNB1	-2.66902	0.000252	***ELANE***	-1.81864	0.017445
ESR2	-2.60176	0.001037	***IL10***	-1.52397	0.01986
EBI3	-2.71193	0.003676	***MME***	-1.82379	0.021736
MGMT	-1.6069	0.00496	***CD163***	-2.2306	0.023022
C4BPA	-2.37316	0.006365	***CD200***	-1.91969	0.031294
CD70	-2.40974	0.00684	***PDGFRA***	-2.06497	0.031907
LOC477699	-2.71261	0.008822	***CXCL13***	-1.54552	0.032276
LOC484343	-1.66527	0.008901	***MASP2***	-1.84317	0.032638
TGFB2	-1.6267	0.009046	***S100A8***	-2.10321	0.035321
CSF2RB	-1.65022	0.011495	***PDPN***	-2.26439	0.043223
MEFV	-2.08317	0.0115	***CD1B***	-2.26539	0.045821
FCRL2	-1.51	0.013555	***LOC102154078***	-2.06142	0.047483
PLAU	-1.71697	0.013683	***TNFSF18***	-1.82191	0.049655
